# Conceptions of Legacy Among People Making Treatment Choices for Serious Illness: Protocol for a Scoping Review

**DOI:** 10.2196/40791

**Published:** 2022-12-09

**Authors:** Marlaine Figueroa Gray, Matthew P Banegas, Nora B Henrikson

**Affiliations:** 1 Kaiser Permanente Washington Health Research Institute Seattle, WA United States; 2 Department of Anthropology University of Washington Seattle, WA United States; 3 Department of Health Systems Science Kaiser Permanente Bernard J Tyson School of Medicine Pasadena, CA United States; 4 Kaiser Permanente Center for Health Research Portland, OR United States; 5 Radiation Medicine and Applied Science School University of California San Diego La Jolla, CA United States; 6 Department of Health Systems and Population Health School of Public Health University of Washington Seattle, WA United States

**Keywords:** communication, clinical decision, end-of-life care-ethics, end of life, palliative, ethic, ethical, psychological care, quality of life, spiritual care, spiritual, legacy, serious illness, critical illness, dying, choice, decision-making, conceptual framework, review methodology, librarian, library science, search strategy, scoping review

## Abstract

**Background:**

Legacy—what one leaves behind and how one hopes to be remembered after death—is an unexplored and important dimension of decision-making for people facing serious illnesses. A preliminary literature review suggests that patients facing serious illness consider legacy when making medical decisions, for example, forgoing expensive treatment with limited or unknown clinical benefit to preserve one’s inheritance for their children. To date, very little is known about the conceptual foundations of legacy. No conceptual frameworks exist that provide a comprehensive understanding of how legacy considerations relate to patient choices about their medical care.

**Objective:**

The objective of this scoping review is to understand the extent and type of research addressing the concept of legacy by people facing serious illness to inform a conceptual framework of legacy and patient treatment choices.

**Methods:**

This protocol follows the guidelines put forth by Levac et al, which expands the framework introduced by Arksey and O’Malley, as well as the Joanna Briggs Institute Reviewer’s manual. This scoping review will explore several electronic databases including PubMed, Medline, CINAHL, Cochrane Library, PsycINFO, and others and will include legacy-specific gray literature, including dissertation research available via ProQuest. An initial search will be conducted in English-language literature from 1990 to the present with selected keywords to identify relevant articles and refine the search strategy. After the search strategy has been finalized, 2 independent reviewers will undertake a 2-part study selection process. In the first step, reviewers will screen article titles and abstracts to identify the eligibility of each article based on predetermined exclusion or inclusion criteria. A third senior reviewer will arbitrate discrepancies regarding inclusions or exclusions. During the second step, the full texts will be screened by 2 reviewers, and only relevant articles will be kept. Relevant study data will be extracted, collated, and charted to summarize the key findings related to the construct of legacy.

**Results:**

This study will identify how people facing serious illness define legacy, and how their thinking about legacy impacts the choices they make about their medical treatments. We will note gaps in the literature base. The findings of this study will inform a conceptual model that outlines how ideas about legacy impact the patient’s treatment choices. The results of this study will be submitted to an indexed journal.

**Conclusions:**

Very little is known about the role of legacy in the treatment decisions of patients across the continuum of serious illness. In particular, no comprehensive conceptual model exists that would provide an understanding of how legacy is considered by people making decisions about their care during serious illness. This study will be among the first to construct a conceptual model detailing how considerations of legacy impact medical decision-making for people facing or living with serious illnesses.

**International Registered Report Identifier (IRRID):**

DERR1-10.2196/40791

## Introduction

### Overview

Legacy—what one leaves behind and how one hopes to be remembered after death—is an unexplored and important dimension of decision-making for people with serious illness. Reflecting on one’s values and legacy when living with a serious illness can provide a heightened sense of dignity, purpose, and meaning, as well as improvement in depressive symptoms and quality of life [1-3]. Such reflection may also provide clarity regarding medical decisions and reduce decisional regret [1,2,4]. Although actions concerning legacy may be taken at any timepoint along an illness experience, legacy work, when undertaken, is often incorporated into end-of-life care or palliative care, and such interventions have been shown to promote emotional and spiritual care of advanced cancer patients [1,5-8].

Based on a preliminary literature review and previous research [9], we are exploring the conceptual foundations of legacy. We conceptualize 3 types of legacy: primary, secondary, and tertiary. Primary legacy includes a living person’s considerations of how they would like to be remembered after death, as well as what artifacts they intentionally leave behind. This may include various types of material and social artifacts, such as financial and legal documents, professional products, and items created for the purpose of memory and social continuity for loved ones [3,10,11]. Planning one’s legacy can be an adaptive process or rite of passage [12] for people living with serious illness [13-15] and may include decisions about medical care. We define secondary legacy as the manner in which others remember a loved one or family member, including bereavement [16] and memorialization [17-19] activities initiated after a person’s death. We term the recognition of the legacy of international or national [20], political [21], or professional impact [22] of a public person not necessarily personally known to those memorializing them to be tertiary legacy.

To date, little research has been conducted on the concept of primary legacy, despite a wealth of scholarship on bereavement and other secondary and tertiary legacy activities. The extant literature on primary legacy typically examines interventions that might include the creation of a legacy document, such as dignity therapy or life review, or how various material artifacts, such as Physician Orders for Life-Sustaining Treatment (POLST), can be created and used, and their impact on patients’ understanding of their illness and preparation for death. We note, in particular, the foundational contribution Boles and Jones [23] offer in their systematic review of legacy interventions for children and adults receiving palliative care [24].

However, how patients define legacy, what it means to them, and how that meaning informs medical decisions are not well understood. A preliminary literature review suggests that people facing serious illness such as cancer consider legacy when making medical decisions, for example, forgoing expensive treatment with limited or unknown clinical benefit to preserve one’s inheritance for their children [25,26]. Yet, very little is known about the role of legacy in the treatment decisions of patients across the continuum of serious illness, from receiving genetic test results that indicate a predisposition to serious illness to receiving a life-limiting diagnosis to choosing treatment options for end-of-life care [27,28]. In particular, no comprehensive conceptual model exists that would provide an understanding of how legacy is considered by people facing serious illness.

### Objective of Conducting the Scoping Review

The objective of this scoping review is to inform a conceptual framework of primary legacy and patient treatment choices by understanding the extent and type of academic discourse, addressing the concept of legacy by people facing serious illness. This scoping review will examine the conceptions of primary legacy as it relates to medical decision-making, excluding literature discussing secondary legacy.

We conducted a preliminary search of MEDLINE, the Cochrane Database of Systematic Reviews, and JBI Evidence Synthesis, and identified no current or in-process systematic reviews or scoping reviews on the topic of legacy and treatment choices of patients. This scoping review will describe the current literature base and identify research gaps. The results will inform a conceptual model of legacy and medical decision-making that will guide future research.

## Methods

### Protocol Design

This protocol follows the guidelines put forth by Levac et al [29], which expands the framework introduced by Arksey and O’Malley [30], as well as the Joanna Briggs Institute Reviewer’s manual [31]. This protocol and the future scoping review are reported in accordance with the PRIMSA-ScR (Preferred Reporting Items for Systematic Reviews and Meta-Analysis Extension for Scoping Review) guidelines [32]. We describe the protocol for this scoping review according to these 6 stages: (1) identification of the research question; (2) identification of relevant studies; (3) selection of eligible studies; (4) charting the data; (5) collating, summarizing, and reporting of the results; and (6) consultation with stakeholders in order to identify additional references about potential studies to include and to collect feedback about the findings uncovered by the review.

### Stage 1: Identifying the Research Question

Through preliminary literature reviews, the research questions this scoping review seeks to answer are (1) how is legacy conceptualized by people facing serious illness? and (2) how is legacy conceptualized during medical care decisions by people facing serious illness?

### Stage 2: Identifying Relevant Studies (Inclusion Criteria)

This review will follow the population, concept, and context framework put forth by the Joanna Briggs Institute [33,34]. The population we will investigate are people facing serious illness. The concept of interest for this scoping review is articles that discuss, directly or indirectly, how people want to be remembered after their own death. This review will not discuss articles not related to illness or medical care, or articles discussing the legacy of another person after death (ie, secondary legacy). As we are primarily concerned with the concept of legacy, this study will exclude intervention, effectiveness, and feasibility studies unless they include a rich qualitative component that speaks to the concept of legacy. The context for this review is open, and sources of evidence relating to any contextual setting are eligible for inclusion [34].

Our preliminary literature review confirms that the concept of legacy is discussed across various disciplines. Given the multidisciplinary sources of evidence, we want to ensure comprehensiveness in literature sources and will include a variety of relevant literature databases. We will explore several electronic databases, including PubMed, MEDLINE, CINAHL, Cochrane Library, PsycINFO, and others, to be informed by the subject matter expert (SME) librarian. We will also hand search the gray literature to identify highly relevant sources, such as reports, and evidence-based legacy programs. Gray literature sources include dissertations (to be accessed via ProQuest) and letters to the editor. We will include empirical articles written in English from 1990 to the present. This time period was chosen in consultation with an SME expert to reflect significant shifts in the provision of hospice and palliative care that provided multiple treatment options for people facing serious illness [35]. Our inclusion and exclusion criteria are represented in Textbox 1.

**Inclusion and exclusion criteria for the scoping review.**

**Inclusion criteria**

- Population
  People with or facing serious illness, such as people with a known family history of disease or people who have experienced a health scare, with a priority focus on historically underserved or vulnerable populations
- Concept/study focus
  Articles that discuss, directly or indirectly, how people want to be remembered after their own death (primary legacy)
  Articles that discuss how people consider legacy when making treatment choices
- Study designs
  Empiric studies, conceptual scholarship, and opinion pieces. Priority focus on studies with relevant qualitative components
- Literature sources
  Priority sources include peer-reviewed books and journal articles
  Legacy-specific gray literature—reports, white papers, etc
  Evidence-based legacy programs are included
- Timing of search
  1990 to the present
- Language
  English

**Exclusion criteria**

- Population
  Clinicians and care team members
  Caregivers only (studies that include both patient and caregiver perspectives will be included)

Note: Population limitations may not be relevant for conceptual or humanities pieces

- Concept/study focus
  Articles discussing the legacy of another person after their death (secondary or tertiary legacy).
  Articles focusing on legacy as a component of bereavement
- Study designs
  None. Lower priority focus on intervention effectiveness and feasibility studies

### Stage 3: Search Strategy and Study Selection

After conducting a preliminary exploration of the academic literature, noting search terms associated with highly relevant articles, and consulting a university SME librarian, we have designed a preliminary search strategy. An initial search will be conducted in English-language literature from 1990 to the present with selected keywords to identify relevant articles and refine the search strategy using MEDLINE/PubMed, CINAHL, PsycInfo, SocialWork, AnthropologyPlus, Web of Science, ProQuest, and Embase databases. We have limited this time window on the guidance of an SME librarian to reflect substantial cultural changes in the United States in end-of-life care. After the search strategy has been finalized, piloted, and conducted, we will undertake a 2-part study selection process. In the first step, 2 independent reviewers will use an electronic abstract screening tool for abstract and full-text review to assess the eligibility of each article based on predetermined exclusion or inclusion criteria. Discrepancies regarding eligibility will be resolved by consensus or consultation with a third team member.

After completing the abstract review, 2 team members will review the full text of articles identified as potentially relevant using the same dual approach, noting reasons for exclusion. Relevant study data will be extracted, collated, and charted to summarize the key findings related to the construct of legacy. To further seek completeness, we will examine the reference lists of highly relevant papers and hand search the gray literature for potentially relevant articles. We will describe the literature flow using the PRISMA (Preferred Reporting Item for Systematic Reviews and Meta-Analyses) literature flow diagram [36,37].

### Stage 4: Preliminary Charting Elements and Associated Questions

Based on the preliminary table of charting elements adapted from Gilfoyle et al [38], we will develop a data abstraction tool (Textbox 2). The team will pilot the tool using a small sample of up to 5 included studies, iteratively refining as needed before proceeding with full abstraction.

For each study included in the review, we will conduct a dual nonindependent review, in which one reviewer will abstract data, and a second reviewer will check for accuracy and completeness. We will use the data abstraction tool developed by the reviewers. A preliminary list of the data to be abstracted is shown in Textbox 2. Abstracted data will include participants, concept, context, study methods, and key findings relevant to the review questions. Any questions that arise from a reviewer will be resolved through additional reviews by one or more team members to check for accuracy and completeness. We may contact the authors of papers to request missing or additional data. We will include the final data abstraction form with the completed review.

**Preliminary table of charting elements and associated questions for data abstraction.**

**Publication details**

- Author
- Years of data collection
- Year of publication
- Country of origin
- Publication type
- Whether publication is open access

**Study characteristics**

- Funder
- Research question
- Discipline
- Aims/purpose
- Methodological design
- Study population and demographics (eg, age)
- Disease state
- Disease progression
- Sample size and response rate
- Recruitment approach
- Study context (eg, oncology or hospice)
- Methods (eg, interview, focus group, or intervention)

**Intervention type (if applicable)**

- Perspective
  From what perspective is research presented? (eg, Patient voices directly or commentary from the author?)

**Findings**

- Definition of legacy
  What terms and keywords do the authors use to define legacy?
- Legacy concepts/constructs
  What concepts or constructs are included?
- Theoretical frameworks
  What theoretical/epistemological frameworks inform this study?
- Care context
  What care context does the study examine?
- Treatment choices
  How is legacy considered in treatment decision-making?
- Material and social artifacts
  What items, values, or types of artifacts do people leave behind for the purposes of legacy?
- Social milieu
  What aspects of a person’s social milieu are discussed?
- Practical steps in creating a legacy
  How is legacy discussed in terms of people’s labor?
- Legacy tension
  What types of tension regarding legacy are discussed?
- How death relates to legacy
  Was type and manner of death discussed as impacting or contributing to legacy?
- Social personhood
  How is social personhood discussed in the context of legacy? (eg, how do people think about continuing as a social presence in people’s lives after they die?)

**Author conclusions**

- What recommendations are made by the author?

**Study limitations/applicability**

- What are the limitations in study design, population, or approach that limit interpretation applicability for the scoping review?

### Stage 5: Collating, Summarizing, and Reporting the Results

After abstracting data, the team will review the data and identify themes related to the research questions. We will report findings using a combination of tables and diagrammatic representations. Narrative summaries will accompany each result, describing how the results relate to the research questions, including any unexpected or particularly notable findings. We will comment on any gaps observed in the literature base, research needs, and implications for practice. We will synthesize the findings into the final conceptual model.

### Stage 6: Consultation with Knowledge Users

As put forth by Levac et al [29], consultation with stakeholders is an important element of methodological rigor in scoping reviews. We will share preliminary findings from stage 5 with stakeholders and incorporate their expertise and perspective [29]. We will map findings from these conversations to conceptual domains through the active collaboration of stakeholders from the community, health services, and academic sectors.

### Ethical Considerations

This scoping review consists of reviewing and collecting data from publicly available materials and as such does not require ethics approval.

## Results

This study will identify how people facing serious illness conceptualize legacy, and how their thinking about legacy impacts the choices they make about their medical treatments. We will describe our literature flow using the PRISMA flow diagram [36]. We will present data extracted via charts and tables and narratively describe the results, noting gaps in the literature base. We will provide a discussion of their significance and present a conceptual model outlining how legacy motivations impact health-related treatment choices.

This study is funded by the National Cancer Institute. We began this scoping review in February 2022 and plan a manuscript submission in late 2022 or early 2023 to an indexed journal.

## Discussion

### Summary

This paper describes the protocol for a planned scoping literature review. Very little is known about the role of legacy in the treatment decisions of patients across the continuum of serious illness. In particular, no comprehensive conceptual model exists that would provide an understanding of how legacy is considered by people making decisions about their care during serious illness. This study will include a scoping review of major research databases to develop a conceptual model that can inform future studies and interventions that investigate the role of legacy in medical decision-making. This scoping review protocol adheres to Levac et al’s [29] guidelines, building on Arksey and O’Malley’s [30] framework, and to the methods manual from the Joanna Briggs Institute.

This scoping review contains important strengths. It is embedded in an established health research partnership and will include the involvement of coresearchers from multiple sites with diverse expertise in the analysis and interpretation stages. This scoping review includes multiple reviewers for all phases of identification and selection. This scoping review has a priority focus on historically underserved or vulnerable populations. This scoping review is limited to English-language articles published from 1990 to the present; translation of non-English language articles is not feasible for this review. This represents a potential limitation and may result in some missed articles. However, although the formal literature search is a limited time period, we intend to include seminal or highly relevant articles identified through hand searching. We also intend to prioritize the inclusion of research with participants who are non-English speakers.

### Conclusion

This study will be among the first to construct a conceptual model detailing how considerations of legacy impact medical decision-making for people facing or living with serious illness.

## Abbreviations

POLST: physician orders for life-sustaining treatment
PRISMA: Preferred Reporting Item for Systematic Reviews and Meta-Analyses
PRISMA-ScR: Preferred Reporting Items for Systematic Reviews and Meta-Analyses Extension for Scoping Reviews
SME: subject matter expert

## Data Availability

A list of included articles used in the scoping review this protocol outlines will be published in future work detailing the results of the scoping review.
